# A Time-Domain Reflectometry Method with Variable Needle Pulse Width for Measuring the Dielectric Properties of Materials

**DOI:** 10.3390/s16020191

**Published:** 2016-02-04

**Authors:** Andrzej Wilczek, Agnieszka Szypłowska, Marcin Kafarski, Wojciech Skierucha

**Affiliations:** Bohdan Dobrzański Institute of Agrophysics of the Polish Academy of Sciences, Doświadczalna 4, 20-290 Lublin, Poland; a.szyplowska@ipan.lublin.pl (A.S.); m.kafarski@ipan.lublin.pl (M.K.); w.skierucha@ipan.lublin.pl (W.S.)

**Keywords:** time-domain reflectometry (TDR), dielectric permittivity, relaxation time, Debye model, FDTD simulations

## Abstract

Time-domain reflectometry (TDR) methods used for measuring the dielectric properties of materials mostly utilize step or needle electrical pulses of constant amplitudes and shapes. Our novel approach enables determining the dielectric relaxation time of a sample using the analysis of the amplitudes of reflected pulses of two widths, in addition to bulk dielectric permittivity and electrical conductivity commonly obtained by the TDR technique. The method was developed for various values of electrical conductivity and relaxation time using numerical simulations of a five-rod probe placed in a material with complex dielectric permittivity described by the Debye model with an added electrical conductivity term. The characterization of amplitudes of two pulses of selected widths was done with regard to the dielectric parameters of simulated materials. The required probe parameters were obtained solely from numerical simulations. Verification was performed for the probe placed in aqueous KCl solutions with 14 different electrical conductivity values. The determined relaxation time remained roughly constant and independent of electrical conductivity. The obtained electrical conductivity agreed with the reference values. Our results indicate that the relaxation time, dielectric permittivity and electrical conductivity of the tested solutions can be simultaneously determined using a simple analysis of the amplitude and reflection time of two needle pulses of different widths.

## 1. Introduction

The dielectric permittivity of materials is a complex value. The real part is mainly associated with the effects of molecular polarization, and the imaginary part represents the loss of energy necessary to force the polarization and conduction state. Electron and atomic polarizations occur in solids, whereas in polar liquids orientation polarization dominates. Electrical conductivity, induced by ionic conductivity, is found in liquids [1,2]. The additional phenomenon of polarization resulting from the interaction of solid and liquid particles depending on their volume fraction, chemical composition, dielectric properties of component mixtures, appears in porous bodies like mixtures of the abovementioned materials with air [3,4]. Dielectric spectroscopy identifies the mechanisms of dielectric dispersion in biological materials, liquid and semi-liquid mixtures and also in materials like agricultural products, soil, etc. The complex dielectric permittivity (CDP) spectrum and its description with the selected model enables us to gain more knowledge about the physical and chemical properties of the tested materials [5,6], and also can be used in assessing their quality [7,8]. The temperature variation of the spectrum has also been studied, and requires a comprehensive description of the measurement under various thermal conditions [9,10,11,12].

Dielectric permittivity measurements are used in many areas such as environmental protection, monitoring of moisture [13,14] and other soil properties, as well as the interpretation and calibration of remote (satellite) soil moisture measurements [14,15,16,17,18], studies of infiltration of water (and solutes) in soil [19,20], quality control of food and agricultural products [21,22], agrophysics [20,23] and other areas related to agriculture [7]. Due the scale effect, heterogeneous granular materials require measurements of a bigger volume than is sufficient for homogeneous materials [24]. For this reason, the TDR measurement method using multi-rod probes for broadband determination of dielectric properties of materials is still under study. 

The CDP spectrum of materials with dispersive properties is described by many models with different numbers of relaxation times, and an additional term for conductivity [3,25]. A TDR signal is commonly applied as a step or a needle pulse. Analysis of pulse amplitude and its reflection time from the end of the probe allows the simultaneous determination of the bulk dielectric permittivity and the electrical conductivity of the tested material [26,27,28]. The dependence of the step pulse rise time of the reflected TDR signal on the material’s dielectric dispersion and electrical conductivity [29] was observed. However, this dependence is not commonly used for the assessment of the dielectric relaxation time of the tested material. The determination of the CDP spectrum from TDR measurements consists of solving the inverse problem in various ways. For this purpose, a multi-section transmission lines method [30], or the Fourier analysis of signals is used. The model parameters describing the CDP spectrum are matched to the TDR signal obtained for a given probe model [31,32]. The CDP spectrum of porous and granular materials is also determined by means of transmission coaxial cells connected to a vector network analyzer (VNA) [33]. The measurement needs a specified volume of test material being placed and sealed inside the cell. Currently, there are no simple methods for measuring the parameters of the CDP spectrum that can be applied in field monitoring systems using multi-rod probes easily installed in porous granular materials such as soil. 

This work presents a variable needle pulse width method of dielectric relaxation time and electrical conductivity determination for applications in the measurement of the dielectric properties of materials. To date, the TDR pulse technique with a modified width has not been applied for this purpose. Commercially available TDR devices use a pulse of fixed parameters selected by the device manufacturer based on the length and geometry of the applied probe [14]. The proposed methodology for determining functional dependencies of TDR needle pulse amplitude on dielectric properties of the material under test was based solely on computer simulations and selected signal processing methods. The aim of this work is to verify the hypothesis that it is possible to simultaneously determine the dielectric permittivity, electrical conductivity σ and the relaxation time τ of the measured material based on the analysis of the time between the incident and reflected pulses and their amplitudes, provided that the initial TDR pulses are of different width. Electrical conductivity, dielectric permittivity and the relaxation time all influence the amplitude of TDR pulses. Nevertheless, the verification performed on aqueous KCl solutions demonstrated that the proposed method enables selective determination of τ and σ for a material of given dielectric permittivity.

## 2. Materials and Methods

### 2.1. Overview of the Method 

**Figure 1:**
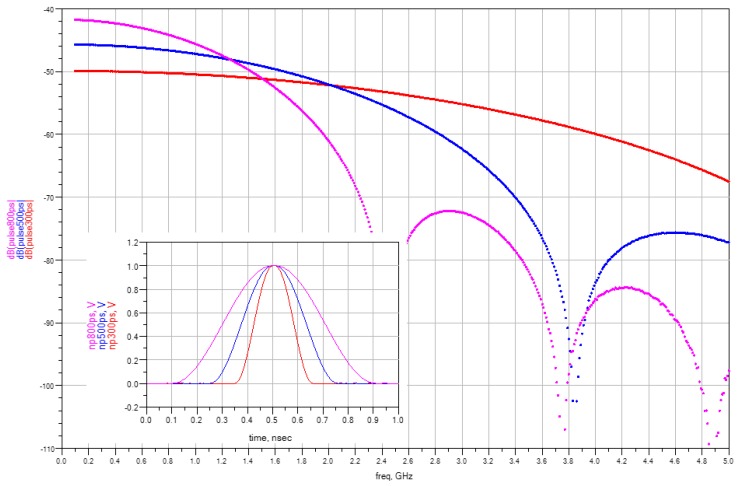
The spectra of 300–800 ps width needle pulses (repetition period = 100 ns).

**Figure 2:**
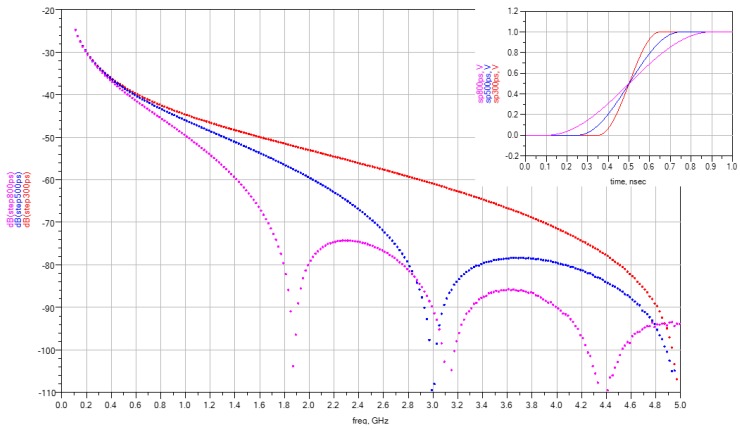
The spectra of 300–800 ps rise-time step pulses (repetition period = 100 ns, duty cycle = 50%).

The spectral characteristics of the TDR pulses depend on their shape. Figure 1 and Figure 2 show the envelope of discrete spectra of commonly used TDR needle measurement signals obtained using Advanced Design Studio (ADS) software [34] from Keysight (Santa Rosa, CA, USA, formerly Agilent) for needle pulse widths and step rise times of 300 ps, 500 ps and 800 ps, respectively. The spectra were obtained using a chirp Z-transform for the same pulse repetition frequency of 10 MHz. In signal processing theory [35], it is known that the amplitude and the spectra density change with the pulse repetition frequency, while the spectra envelope shape remains unchanged. Figure 1 shows the comparison of spectra for needle pulses of different width. The amplitude difference of the components at the frequency of 100 MHz is 8 dB for the two extreme pulse widths, while the amplitude components are almost equal at about 1.5 GHz. In the case of a step pulse, the component amplitudes begin to differentiate with increasing frequency, wherein for the lowest analyzed frequencies they remain constant irrespective of the various rise times (Figure 2).

The granular porous material, having dielectric dispersion, acts as a low-pass filter for the TDR signal. As a result, the shape of the pulse reflected from the end of the rods varies with the value of the CDP imaginary part, and its changes with frequency. For dispersive materials, the character of the step pulse spectrum translates into decrease in rise time, but negligible amplitude dependency while measuring the reflected signals of various initial rise times. On the other hand, the amplitude of the reflected needle pulses heavily depends on the initial pulse width.

**Figure 3:**
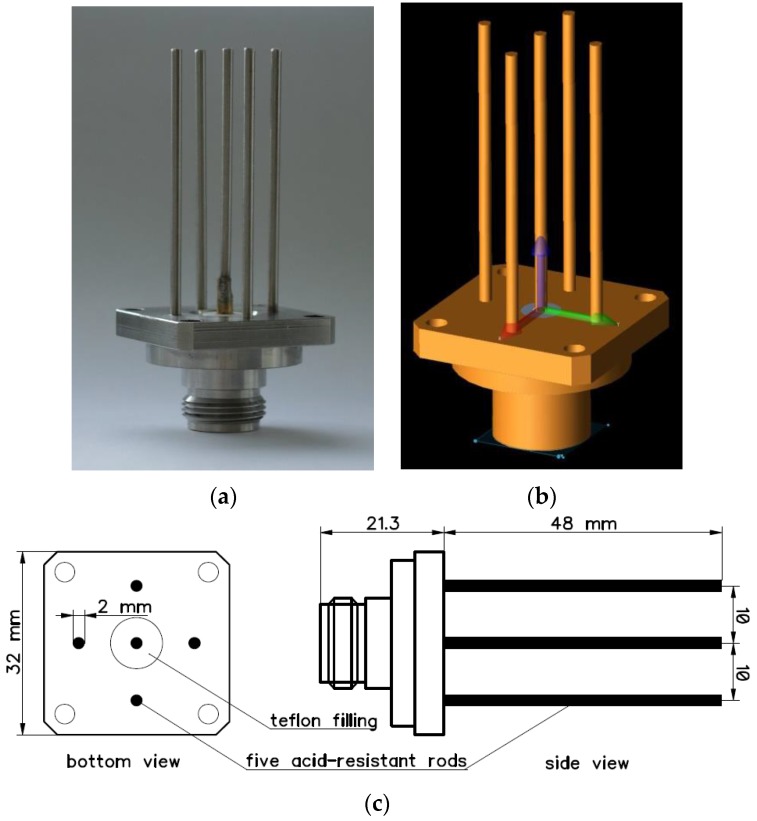
(**a**) Photo of the applied five-rod probe; (**b**) Simulation model; (**c**) Probe dimensions.

Changing the spectral characteristics of signals by adjusting the needle pulse width enables one to determine the parameters of the selected dielectric model of the measured material. This is accomplished by the application of functional relationships between the reflected pulse shape characteristics and dielectric properties of the material under test, determined from the simulations of variable width needle pulses reflected from the TDR probe end. In practical application it is necessary to use a needle pulse generator in the TDR meter, with a controllable pulse width that is the subject of a patent application [36]. The simulations and subsequent experimental verification were carried out with the use of a five-rod probe, made of four press-fitted stainless steel rods and the fifth rod soldered to the inner pin (Figure 3) of the commercially available N-type connector (R161422120, Radiall, Paris, France). The number of external rods was selected out of convenience in order to decrease leaking of electric field outside of the probe. Two- or three-rod configurations would require larger sample volumes in order to avoid influence of the sample container during verification measurements.

**Figure 4:**
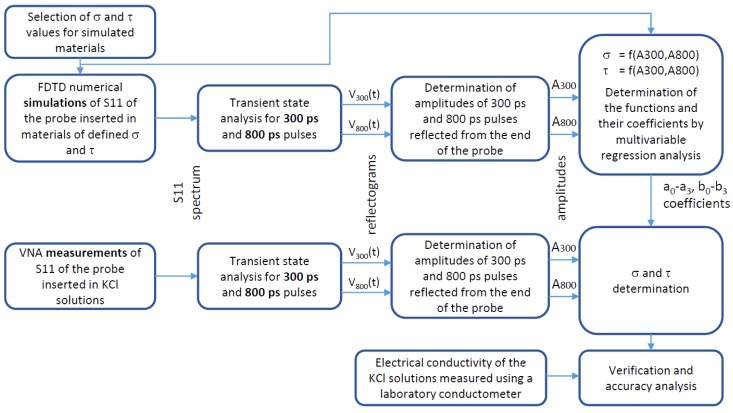
The workflow diagram of the presented study.

The workflow diagram of the development and verification of the method under study is presented in Figure 4. Firstly, numerical simulations of S11 parameters of the probe placed in materials of chosen dielectric properties were performed. Then, the reflectograms for TDR needle pulses of two selected widths were obtained. Using multivariate non-linear regression analysis, the dependencies of the pulses’ amplitudes (see Figure 7 below) on the electrical conductivity and dielectric properties of the studied materials were obtained. Next, the measurements of S11 parameters of selected materials using a VNA were performed. On the basis of the measured S11 parameters and the functional dependencies of the pulses’ amplitudes on the materials’ properties, the values of electrical conductivity and relaxation time for each measured sample were obtained. These values were then used to assess the accuracy of the presented method.

### 2.2. Computer Simulations and Transient State Analysis 

The presented method was based on FDTD numerical simulations performed in order to obtain S11 parameter values for the probe model, with a geometry reflecting the actual probes used in measurements (Figure 3). The S11 simulation results describe the probe response when inserted into a dielectric material. These frequency-dependent data were used for the subsequent temporal analysis of transient states performed in order to obtain reflectograms for needle pulses of two widths.

The Debye model (Equation (1)) [37] was used for the description of the complex dielectric permittivity of the material filling the probe:

(1) $$\varepsilon^{*}\left(\omega \right)=\varepsilon_{\infty }+\frac{\varepsilon_{s}-\varepsilon_{\infty }}{1+j\omega \tau }-\frac{j\sigma }{\omega \varepsilon_{0}}\;\mathrm{where}\;\omega =2\pi f$$

**Figure 5:**
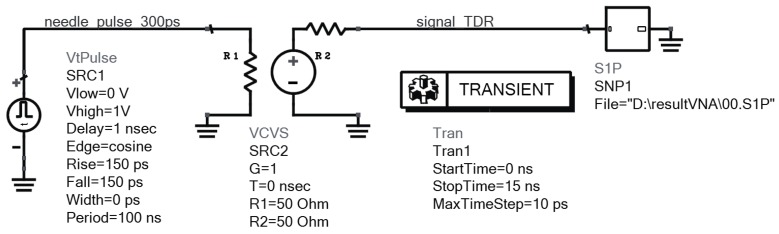
Connection diagram of the circuit elements in the ADS program for signal analysis in the time domain for the S11 spectrum.

The value *ε_s_* can be determined by measuring the TDR pulse propagation time. The present study focused on the influence of electrical conductivity and dielectric relaxation time. Therefore, the simulations were performed for an arbitrary material with constant values *ε_∞_* = 5 and *ε_s_* = 80 (as for water) and of various relaxation times *τ* and electrical conductivity *σ*. The probe model simulations were performed using the electromagnetic professional (EMPro) [38] software design platform of the Keysight electronic design automation. Simulations were carried out in the 20 MHz–8 GHz frequency range, which resulted in obtaining 143 files containing the complex S11 parameter values for 11 cases of electrical conductivity *σ* in the range of 0–0.5 S·m^−1^, and 13 relaxation time values in the range of 1–13 ps. Subsequently, in each case, the analysis of transient states was carried out in the time domain. Keysight ADS software design platform was used for this analysis, where two types of TDR needle pulse inputs of 1 V amplitude, 300 ps and 800 ps width, were applied. The widths of the pulses were selected as such in order to provide significant amplitude difference, while maintaining good resolution and sensitivity for a given probe rods length. Figure 5 shows a connection diagram for signal analysis in the time domain.

The ADS analysis produced reflectograms showing the time and amplitude of reflections from the five-rod probe, as the response to two initial needle pulses of different width.

### 2.3. Verification Measurements 

The material tested was a series of KCl aqueous salt solutions with 14 different electrical conductivity values in the range of 50 µS·m^−1^ to 0.5 S·m^−1^ at a constant temperature of 22 ± 0.35 °C. Measurements of the S11 parameters were carried out by a vector network analyzer of the type ZVCE (Rohde & Schwarz, Munich, Germany) in the frequency range 20 kHz–8 GHz, with the attached five-rod probe (Figure 3). The salt solutions were prepared by adding small amounts of KCl to distilled water and measuring the electrical conductivity using a conductometer CX-701 (Elmetron, Zabrze, Poland). For the VNA measurements, the solutions were poured into a cylindrical glass beaker of 95 mm height and 70 mm in diameter. The S11 parameters from VNA served as the input data to the ADS software, which performed the respective analysis in the time domain of the 5-rod probe response to the input pulses.

## 3. Results and Discussion

### 3.1. Numerical Simulations and the Derivation of the Functional Dependencies 

**Figure 6:**
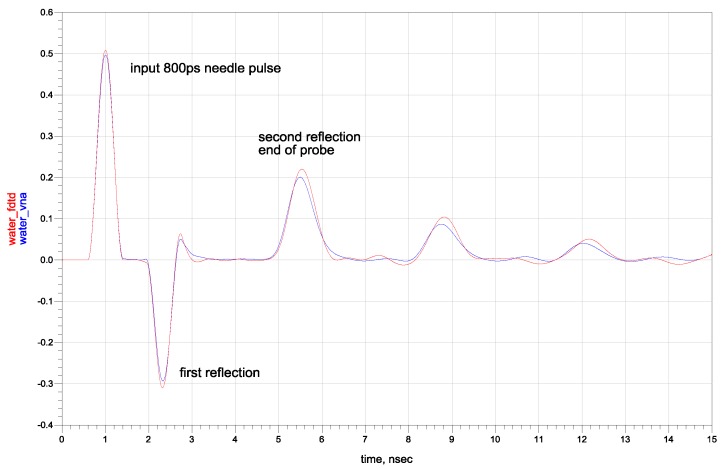
The comparison in the time domain of the results obtained with VNA S11 measurements of distilled water (the blue curve) and the S11 simulated (the red curve) for the five-rod probe inserted into material with Debye parameters: *ε_∞_* = 5, *ε_s_* = 80, *σ* = 50 µS·m^−1^, *τ* = 8 ps.

Firstly, the conformity between the simulations and the measurement results was assessed. The simulation results and transient analysis showed good amplitude-time agreement with the results obtained from the real measurements of the five-rod probe connected to the VNA, as can be seen for the case of distilled water in Figure 6.

**Figure 7:**
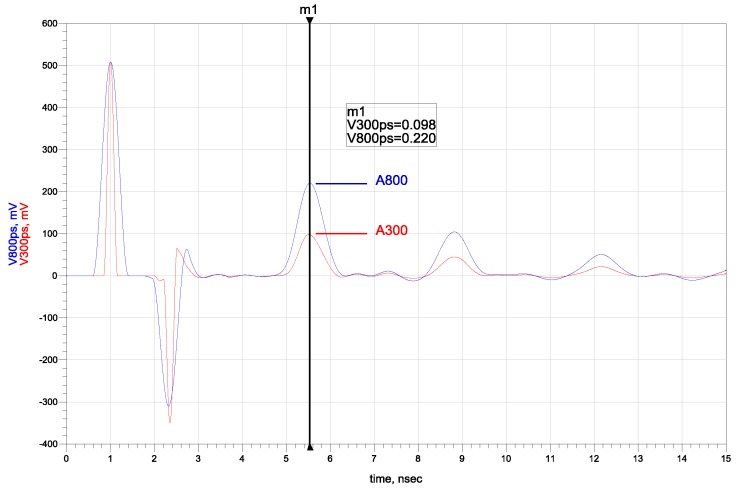
The time-domain results for needle pulses of 300 ps and 800 ps width for simulated Debye material characterized by *ε_∞_* = 5, *ε_s_* = 80, *σ* = 50 µS·m^−1^, *τ* = 8 ps.

**Figure 8:**
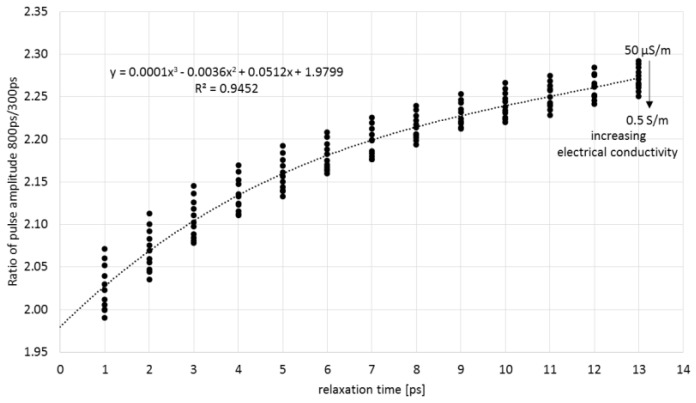
The relationship between the amplitude ratio of 800 ps and 300 ps pulses and relaxation time for simulation parameters: *ε_∞_* = 5, *ε_s_* = 80, *σ* ∈ [50 µS·m^−1^, 0.5 S·m^−1^].

The analysis of the reflectograms obtained for two needle pulse widths for the simulated materials showed that the amplitude of the signal reflected from the probe ending increased with the initial needle pulse width for materials with a non-zero relaxation time (Figure 7). The simulation results are in agreement with the measured data. The obtained time and amplitude values of pulses of two chosen widths were correlated to the input simulation parameters of the electrical conductivity *σ* and relaxation time *τ*. The dependence of the ratio of analyzed pulses’ amplitudes obtained from numerical simulations with respect to the simulated materials’ dielectric relaxation time for various electrical conductivities are presented in Figure 8.

**Figure 9:**
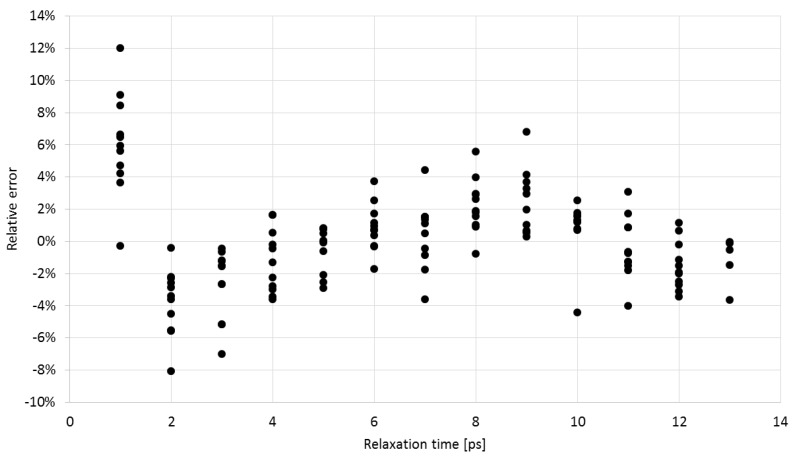
The relaxation time relative error determined from Equation (2) for the simulated materials.

As can be seen, the observed changes are related to the effects of the relaxation time of the material in which the probe is placed. With an increase in the relaxation time, the ratio of the pulse amplitudes increases. Also, the ratio of the pulse amplitudes decreases with an increase in conductivity. However, conductivity has a smaller impact than the relaxation time. It was found that an equation of the form (2) described the best the inverse relation, which allowed to determine the relaxation time of the material from measuring the pulse amplitude ratio *A*_800_/*A*_300_. Figure 9 shows the values of relative error of determining the relaxation time from Equation (2), based on the simulated data. The coefficients a_0_–a_3_ were found by multivariable regression:

(2) $$\tau ={\left(a_{3}{\left(\frac{A_{800}}{A_{300}}\right)}^{2}+a_{2}\frac{A_{800}}{A_{300}}+a_{1}A_{800}+a_{0}\right)}^{2}$$

Knowing the relaxation time and the amplitude of the 800 ps pulse (*A*_800_), it is possible to determine the electrical conductivity of the KCl solution using Equation (3):

(3) $$\sigma =\ln\left(\left(\frac{1}{A_{800}}-b_{3}\tau \right)/b_{2}\left(b_{1}\tau +1\right)\right)/b_{0}$$

**Figure 10:**
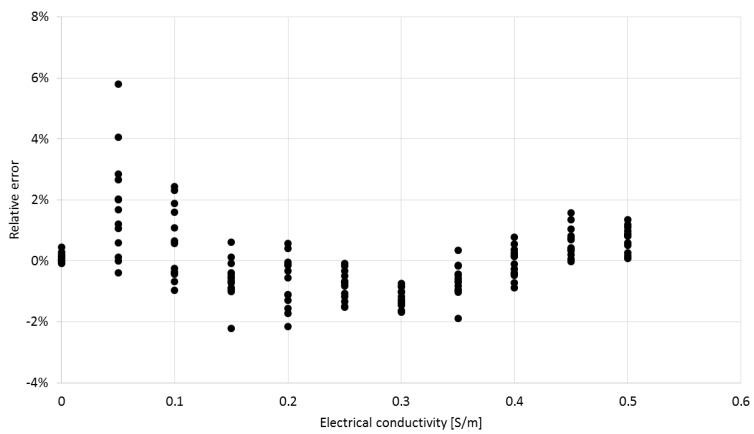
The relative error of electrical conductivity determination from Equation (3) for the simulated materials.

The form of Equation (3) was chosen to best fit the data. The coefficients *b_0_–b_3_* can be determined by multivariable regression. Figure 10 shows the values of relative error of determining the electric conductivity from Equation (3), based on the simulated data.

### 3.2. Verification of the Method on the Measured Solutions 

The obtained functions were used to selectively determine the value of electrical conductivity *σ* and relaxation time from the S11 measurements with the VNA for the 5-rod probe placed in KCl aqueous solutions of 50 µS·m^−1^–0.5 S·m^−1^ conductivity.

**Figure 11:**
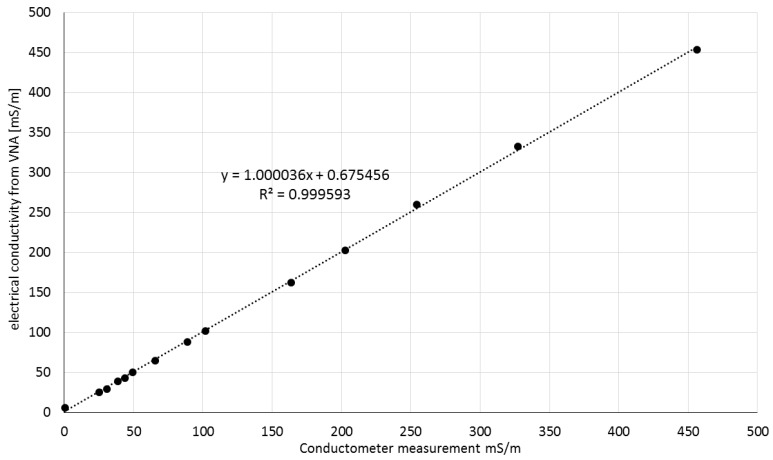
The electrical conductivity values of the VNA-measured solutions, obtained using Equation (3), with respect to the actual electrical conductivity of the solutions measured using a laboratory conductometer.

**Figure 12:**
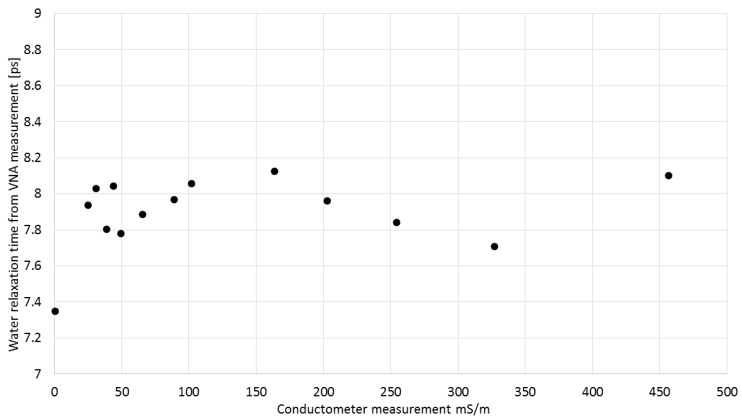
The values of relaxation time obtained using Equation (2) of VNA-measured KCl solutions of various electrical conductivities.

Electrical conductivity values obtained using Equation (3) (Figure 11) were found to be fully compliant with those measured by a conductometer. The relation between these values is described by a linear function with the slope of 1.000036 and R^2^ = 0.9996. Figure 12 presents the water relaxation times of the VNA-measured KCl solutions obtained using Equation (2). The average value of the relaxation time was 7.9 ps with standard deviation 0.2 ps. The relaxation time of pure water at 22 °C should be 8.9 ps [39]. The 1 ps difference is not so big taking into account that the measurement was conducted up to 8 GHz whilst the main relaxation effect is observed at about 20 GHz.

The results shown in Figure 11 and Figure 12 suggested that using the TDR method with a controlled needle pulse width, it is possible to determine simultaneously and selectively the relaxation time, electrical conductivity and bulk dielectric permittivity of salt solutions by means of the presented five-rod probe.

## 4. Conclusions

The presented method enables selective determination of dielectric relaxation time and electrical conductivity of a material using the amplitudes of two TDR needle pulses of different widths. To obtain the functional dependencies necessary for the presented method for one value of dielectric permittivity, just eight function parameters are required (Equations (2) and (3)), which can be obtained from numerical FDTD simulations. In the present study, the parameters enabling determination of electrical conductivity and dielectric relaxation time of a material described by the Debye model with static dielectric permittivity of water were obtained. A comprehensive derivation of the functional dependencies in the entire variability range of dielectric permittivity, necessary for the measurements of arbitrary materials, requires performing further computer simulations. The demonstration of the proposed method on materials with arbitrary dielectric permittivity is planned as a subject of a future study. The presented results showed that it is possible to expand the measurement range of the TDR method with the possibility of determining selectively the dielectric relaxation time and electrical conductivity of a material, what was the aim of the present study.

This method may be applied to studies of a variety of non-homogeneous granular and porous materials where it is impossible to apply the very popular open-ended coaxial probe [40] because of its small volume sensitivity. Example applications of the method presented here could include dielectric measurements of moisture of building materials, quality assessment and control of agricultural materials and products, soil moisture and salinity determination methods of improved accuracy without the need for soil specific calibrations, etc. A simple modification of the TDR technique consisting of using needle pulses of various specific parameters creates opportunities for its widespread use in environmental monitoring systems. Therefore, it will be possible in the future to verify the hypothesis of whether soil texture, or the amount of the component fractions of porous materials, could be assessed on the basis of dielectric parameters determined by the variable needle pulse width TDR method.
